# Rapid AC Electrokinetic Micromixer with Electrically Conductive Sidewalls

**DOI:** 10.3390/mi13010034

**Published:** 2021-12-27

**Authors:** Fang Yang, Wei Zhao, Cuifang Kuang, Guiren Wang

**Affiliations:** 1 Key Laboratory for Molecular Enzymology and Engineering of Ministry of Education, School of Life Sciences, Jilin University, Changchun 130012, China; 2 State Key Laboratory of Photon-Technology in Western China Energy, International Scientific and Technological Cooperation Base of Photoelectric Technology and Functional Materials and Application, Institute of Photonics and Photon-Technology, Northwest University, Xi’an 710127, China; zwbayern@nwu.edu.cn; 3 State Key Laboratory of Modern Optical Instrumentation, Zhejiang University, Hangzhou 310027, China; cfkuang@zju.edu.cn; 4 Department of Mechanical Engineering and Biomedical Engineering Program, University of South Carolina, Columbia, SC 29208, USA

**Keywords:** microfluidics, electrokinetics, mixing, micromixer

## Abstract

We report a quasi T-channel electrokinetics-based micromixer with electrically conductive sidewalls, where the electric field is in the transverse direction of the flow and parallel to the conductivity gradient at the interface between two fluids to be mixed. Mixing results are first compared with another widely studied micromixer configuration, where electrodes are located at the inlet and outlet of the channel with electric field parallel to bulk flow direction but orthogonal to the conductivity gradient at the interface between the two fluids to be mixed. Faster mixing is achieved in the micromixer with conductive sidewalls. Effects of Re numbers, applied AC voltage and frequency, and conductivity ratio of the two fluids to be mixed on mixing results were investigated. The results reveal that the mixing length becomes shorter with low Re number and mixing with increased voltage and decreased frequency. Higher conductivity ratio leads to stronger mixing result. It was also found that, under low conductivity ratio, compared with the case where electrodes are located at the end of the channel, the conductive sidewalls can generate fast mixing at much lower voltage, higher frequency, and lower conductivity ratio. The study of this micromixer could broaden our understanding of electrokinetic phenomena and provide new tools for sample preparation in applications such as organ-on-a-chip where fast mixing is required.

## 1. Introduction

Mixing of two or more fluids is always crucial in the application of microfluidics in chemical engineering, environmental engineering, and even biomedical and biochemical analysis such as enzyme reaction, protein folding, DNA purification, etc. [1]. Fast mixing can generate stronger signals to increase the sensitivity and enable more accurate measurement of chemical reaction kinetics. However, since the flows are mainly laminar in microfluidics, mixing is carried out by molecular diffusion and fast mixing is not easily achieved. Highly efficient and fast mixing of two fluids inside microchannels could be highly challenging. Therefore, developing new techniques and methodologies to increase the interfacial surface area for enhancing the mixing processes is crucial to improve the corresponding performance of ‘lab-on-a-chip’ devices [2].

Many new micromixer techniques have been developed in last two decades [3]. Generally, the ‘micromixer’ can be categorized into two groups: passive and active mixers [4]. Passive mixers do not require external energy. They enhance mixing processes through the augmentation of diffusion through fluid folding, stretching, and tilting by special design of microchannel geometry [5]. In contrast, active mixers usually employ external energy to introduce disturbances on the flow to enhance fluid mixing. Several types of active micromixers with flow disturbances generated in terms of temperature [6], pressure [7], electrohydrodynamics [8,9], acoustics [10], as well as magnetics [11], have already been reported to effectively enhance fluid mixing in microchannels.

Among the aforementioned methods, liquid or particle motion can be effectively manipulated by electrokinetic mechanisms, including electro-osmosis, electrophoresis, dielectrophoresis, and electrowetting, etc., since electric body force (EBF) is more effective on small scales and interfaces [12,13]. In 2001, Oddy et al. [14] presented an active micromixer in which an AC electric field induces a chaotic flow field to enhance the mixing of two pressure-driven flow streams. Moreover, it was demonstrated by Shin et al. [15] that more chaotic trajectories can be generated in a cross-shaped microchannel by a time-dependent electric field. Recently, many works on electrokinetic instability (EKI) were accomplished and attracted much attention, it is a phenomenon described by charge accumulation at perturbed interfaces due to electrical conductivity gradients, which exist in the bulk flow [16,17,18,19,20]. Although many significant results have already been obtained through those previous works [21,22,23,24,25], much effort is still needed to improve our understanding of electrokinetic mixing under AC electric field, to make the electrokinetic micromixers more efficient and flexible for “lab-on-a-chip” applications. 

In 2014, we demonstrated that turbulence [26] and its corresponding ultrafast mixing [27] of two pressure-driven flows can be realized electrokinetically in a microchannel with slightly divergent sidewalls (fabricated with electrodes) at low bulk-flow Reynolds number. However, parallel microchannels are mostly used in microfluidics and the mixing enhancement in the electrically conductive sidewalls in parallel has not been studied. In this paper, we present a parametric study of the rapid fluid mixing inside a T-shaped microchannel, where two streams of pressure-driven flows are disturbed by an externally time-dependent electric field, which is orthogonal to the conductivity gradient at the interface between the two fluids to be mixed. The parameters, such as electrode positions and voltage phase shift between two electrodes, were investigated.

## 2. Materials and Methods

**Figure 1:**
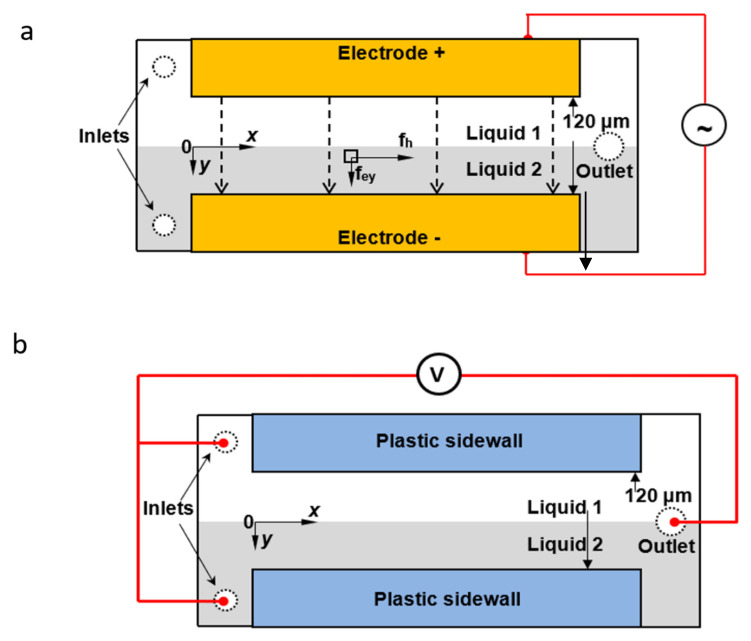
Flow, electrodes configuration, and force descriptions in micromixer. (**a**) Conductive sidewalls micromixer; (**b**) micromixer with electrodes located at the ends of channel.

The schematic of the micromixers is given in Figure 1. Both of the micromixers are T-shaped with parallel sidewalls. Two cases have been considered in this investigation: one has electrically conductive sidewalls, the other has insulated sidewalls with electrodes placed at inlet and outlet. In the former, the sidewalls of the channel are made of gold sheet (as shown in Figure 1a). Here, *x* and *y* denote the streamwise and transverse directions in the main channel, respectively. In the latter, the sidewalls of the micromixer are fabricated with acrylic, as shown in Figure 1b. Platinum electrodes are placed at the inlets and out of the microchannel. The micromixers both have rectangular cross sections of 120 µm in width and 230 µm in height, with the length of 5 mm. Two inlets and one outlet with the diameter of 1 mm were drilled at the ends of the channel. 

Two fluids with different electrical conductivity and permittivity are used for the study. Each fluid enters the micro-fluidic chamber through its own inlet channel. As soon as they contact, a jump in electrical conductivity and/or permittivity is generated at the interface between the two fluids. The flow of an incompressible and Newtonian fluid in presence of an electric field is governed by the Navier–Stokes equations:

(1) $$\rho \left(\frac{\partial \vec{V}}{\partial t}+\vec{V}\cdot \nabla \vec{V}\right)=-\nabla P+\eta \nabla^{2}\vec{V}+{\vec{f}}_{e}$$

where ρ is the fluid density, $\vec{V}\;$denotes the velocity field, *P* refers to the pressure, and η is the (constant) dynamic viscosity. ${\vec{f}}_{e}$ is the EBF attributed to Coulombic force, as:

(2) $${\vec{f}}_{e}=\rho_{f}\vec{E}$$

where $\rho_{f}$ is net charge density which can be expressed as [18]:

(3) $$\rho_{f}=-\frac{\varepsilon \vec{E}\cdot \vec{\nabla }\sigma }{\sigma }$$

where ε is the permittivity of the electrolyte and $\vec{E}$ is the electric field. Due to the presence of electrical conductivity gradients,$\;\vec{\nabla }\sigma$ at interfaces between two streams with different electrical conductivity, which exist in the bulk flow [18,28], non-zero net charge will be accumulated at interfaces when an electric field is applied. Then, an EBF ${\vec{f}}_{e}$ results which distorts the interface of the two fluid streams. If the magnitude of the disturbance is sufficiently large, a transversal convection (secondary flow) can be induced on the interface, destabilize the interface through electrokinetic instability (EKI), and promote the mixing of the two fluids. If the there is no conductivity gradient, which means $\vec{\nabla }\sigma =0$, then no net charge will be induced ($\rho_{f}=0)$, consequently, no body force on liquid will be generated ($\rho_{f}\vec{E}=0),\;$and then no EKI occurs. In this investigation, electric conductivity is not a passive scalar [27] since the EBF can significantly manipulate the flow and accordingly affect the field of electric conductivity.

From Equation (3), it is important to notice that indicated by the term of $\vec{E}\cdot \vec{\nabla }\sigma$*,* the charge density could be minimum when the external electric field is perpendicular to the electrical conductivity gradients, which is the case in that electrodes are placed at the inlets and outlets of the mixing channel. In contrast, when the external electric field is parallel to the electrical conductivity gradients, the charge density can be maximized. In our micromixer, the electrodes are directly used to form the sidewall; therefore, the external electric field is parallel to the electrical conductivity gradients (Figure 1) which will result in a maximum EBF, and strongest distortion between the interface of the two-liquid flow, initially. In the present study, AC voltage signals are used instead of DC voltage due to the fact that bubbles are more easily generated in highly conductive buffer under DC voltage due to electrolysis, which can block the microchannel and thus be detrimental to the performance of microfluidic devices [29]. 

**Figure 2:**
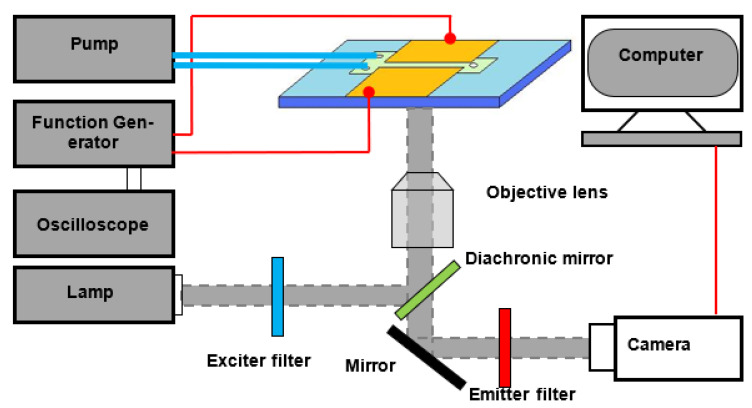
Experimental setup.

A syringe pump (Harvard, Model PHD2000 Programmable, Holliston, MA, USA) was used to pump fluorescent dye solution and DI water from the inlets respectively through the micromixer toward the outlet. Flow visualization were applied to study fluid mixing. Fluorescein sodium salt (C_20_H_10_Na_2_O_5_) was used as the fluorescent dye trace for characterizing the mixing results. Electrically neutral dye rhodamine B (Sigma-Aldrich, Corp., Burlington, MA, USA) was also used as the scalar marker to study conductivity ratio influence on fluid mixing. Phosphate buffer (VWR VW3345-1 pH 7.2) was diluted into DI water as one of the mixing streams to control the conductivity ratio between the two streams. Figure 2 shows the schematic of the experimental setup. The microchip was placed on an inverted fluorescent microscope (Olympus-IX70, Tokyo, Japan) for fluorescence measurements. A function generator (Tektronix, Model AFG3102, Beaverton, OR, USA) was used to apply AC electric signal between these two electrodes.

A mercury lamp was used as the illumination source in the present study. The excitation beam is 488 nm. Upon excitation, the fluorescent solution would emit fluorescence. A 10× objective lens (NA = 0.25) was used for the fluorescence imaging. The fluorescence signal was captured by a sensitive and high-resolution CCD camera (SensiCam QE, PCO, Bavaria, Germany), with an exposure time of 0.1 s. All concentration was quantitatively determined by measuring the fluorescence intensity within each pixel of the camera using MATLAB (MathWorks Inc., Natick, MA, USA). Mixing enhancement results were compared based on concentration profiles of the fluorescent dye along a transverse line that is perpendicular to flow direction of the microchannel at a given streamwise position.

## 3. Results and Discussion

### 3.1. Effect of External Electric Field Direction

To evaluate the influence of directions of external electric field, the mixing results in two cases were compared, e.g., electrodes are placed at the sidewalls (case A) and at the ends of the channel (case B), respectively. The latter has been studied widely as electrokinetic micromixers [18,30]. However, a direct comparison of the two arrangements of the electrodes on mixing has not been carried out before. 

In the experiment, we kept flow rate at 5 µL/min and conductivity ratio of the two streams at 10:1, unless otherwise specified. The external electric fields have strength ($E_{A}$) of both 200 V/cm for the two cases. In this part, a low AC frequency ($f_{AC}=$ 1 Hz) was used.

**Figure 3:**
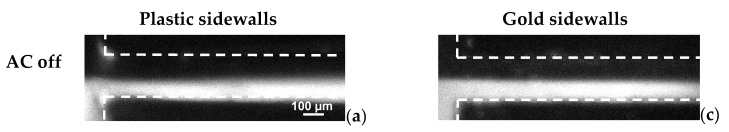

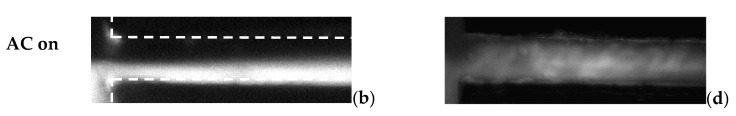
Comparison of mixing results under different electrode positions and corresponding electric fields. In (**a**,**b**), electrodes are located at the ends of the channel (plastic sidewalls) and the electric field is orthogonal to the initial conductivity gradient between the two streams. In (**c**,**d**) electrodes (gold sidewalls) are placed at the sidewalls and the electric field is in transverse direction and in parallel to the initial conductivity gradient between the two streams.

As shown in Figure 3, it is clearly illustrated that under the same $E_{A}$, the mixing is much stronger in case A than case B. For the plastic sidewalls, to achieve $E_{A}=200\;V/\mathrm{cm}$, we had to use a voltage amplifier accompanied with function generator to apply AC voltage on the case B. In this case, it is difficult to apply high AC voltage and high frequency signal simultaneously. However, for the case A, no power amplifier is required to achieve $E_{A}=200\;V/\mathrm{cm}$. The function generator can provide sufficient high $E_{A}$ and AC frequency on the electrodes simultaneously. Figure 3 clearly indicates, as a result of the parallel of electric conductivity gradient and external electric field, that $\vec{E}\cdot \vec{\nabla }\sigma$ reaches maximum. A much larger ${\vec{f}}_{e}$ can be predicted according to Equation (3), relative to that in the case B. In the following section, the electrokinetics micromixer with conductive sidewalls will be characterized.

### 3.2. Effect of Electric Conductivity Ratio

According to Equations (2) and (3), two mixing streams with conductivity gradient were subject to an external electric field; mixing was directly influenced by the ${\vec{f}}_{e}$ [31]. 

In order to conduct a parametric study to quantify the effect of the conductivity ratio of the two streams on the mixing performance, we kept the AC signal of $f_{AC}=$ 10 kHz and $E_{A}$ = 833 V/cm (corresponding to AC amplitude of 10 V_p-p_.) Three conductivity ratios ($\gamma =\sigma_{1}/\sigma_{2}$, with $\sigma_{1}\geq \sigma_{2}$) between the two streams were investigated, and they are 1, 2, and 10, respectively.

**Figure 4:**
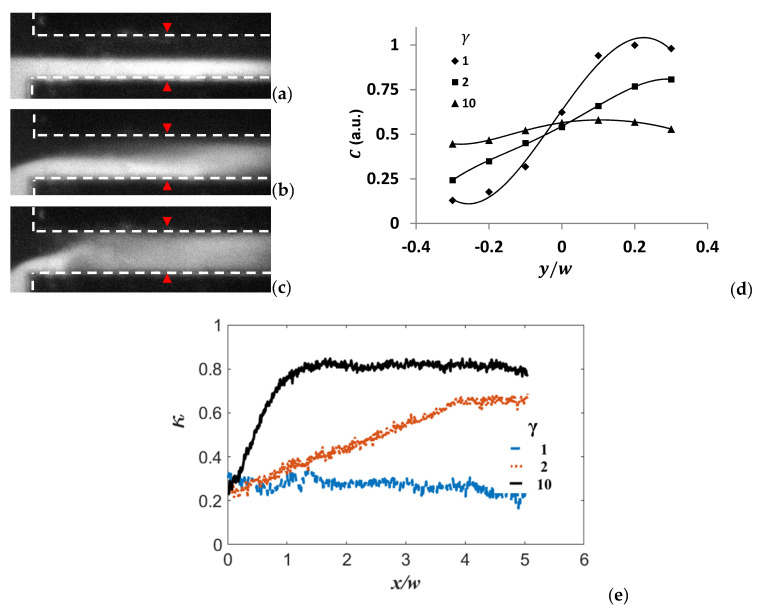
Visualization and comparison of mixing results in the micromixer with different conductivity ratios γ at $E_{A}$ = 833 V/cm. (**a**) γ=1, (**b**) γ=2, (**c**) γ=10, and (**d**) comparison of concentration profile in transverse direction at x/w=3 from the channel entrance (marked by red line) with different conductivity. (**e**) Mixing index varying along streamwise direction at different conductivity ratios.

Mixing performances under different conductivity ratio are shown in Figure 4. Figure 4a,b indicates that the mixing is stronger at γ=2 than that at γ=1. When γ=10 (Figure 4c), the mixing is the strongest among these three cases. In Figure 4d, the corresponding concentration distribution (evaluated by fluorescence intensity) in the transverse direction is displayed. As we know, the stronger the mixing, the more uniform the concentration in the transverse direction at a given streamwise position. The curve should approach flat when the fluids are well mixed in the microchannel. According to Figure 4d, when the conductivity ratio γ is 10, concentration distribution reached a relatively uniform profile at x/w=3 (w is the width of the microchannel) from the entrance. While γ are 1 and 2, at the same streamwise position, C distributions were far away from a flat profile. The mixing result is evaluated by a mixing index κ, which is similar (but different) to the mixing criterion used in Arockiam et al.’s work [32] and is defined as:

(4) $$\kappa =1-\frac{\sqrt{\langle (C-\langle C\rangle {)}^{2}\rangle }}{\langle C\rangle }$$

where 〈·〉 denotes ensemble averaging. Here, 0≤κ≤1. The higher κ, the stronger the mixing. It can be seen from Figure 4e, when γ=1, κ is nearly flat which indicates the mixing is not enhanced under the external electric field. When γ=2, κ increases gradually along streamwise direction. At x/w=4, κ is about 0.65, which is approximately three times larger than that of γ=1. When γ=10, κ increases rapidly and reaches 0.84 at x/w=1.5. It is twice larger than that of γ=2 and the time cost is only 30 ms. Note there is a little fluctuation of κ along streamwise direction, which is because of the non-uniform excitation light distribution of the microscope.

**Figure 5:**
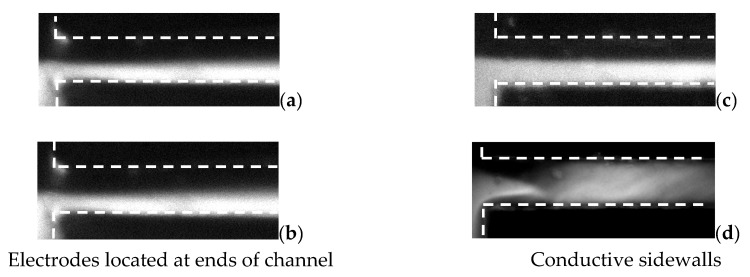
Visualization and comparison of mixing result in micromixer with electrodes located at ends of channel (**a**,**b**) and micromixer with conductive sidewalls (**c**,**d**), (**a**) without voltage, (**c**) with voltage; sidewall, (**b**) without voltage, and (**d**) with voltage.

Specially, mixing results under the same low γ=2 in both conductive sidewalls micromixer and plastic sidewalls micromixer with electrodes located at the ends of the channel were measured and compared, as shown in Figure 5. In this part, the applied electric fields are kept constant, i.e., $E_{A}=$ 200 V/cm for the two mixers. For the case of Figure 5c, a periodic electric field was added to the applied static electric field, to enhance mixing, which is $E_{A}$ = 1667 V/cm (with 20 V_p-p_ voltage) in amplitude and $f_{AC}=$ 10 Hz.

Figure 5 clearly shows that, under electric field $E_{A}=$ 200 V/cm, obviously stronger mixing has been achieved in the micromixer with conductive sidewalls, as shown in Figure 5d. However, in the micromixer with the nonconductive sidewalls, where the electrodes are located at ends of the channel, no obvious mixing enhancement was observed (Figure 5b).

### 3.3. Effect of AC Frequency

Influence of AC frequency on mixing has also been investigated in a wide range from 100 Hz to 2 MHz. Fluid mixing under DC voltage was also presented as a comparison. To study the effect of AC frequency on mixing, the electric field was kept constant as well as in the DC situation, i.e., $E_{A}$ = 1000 V/cm. 

**Figure 6:**
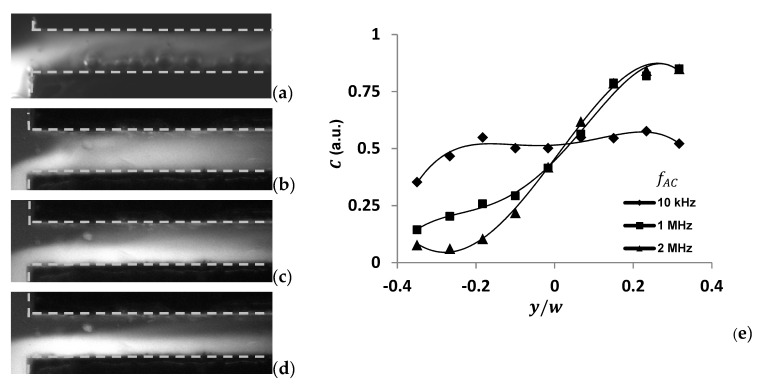

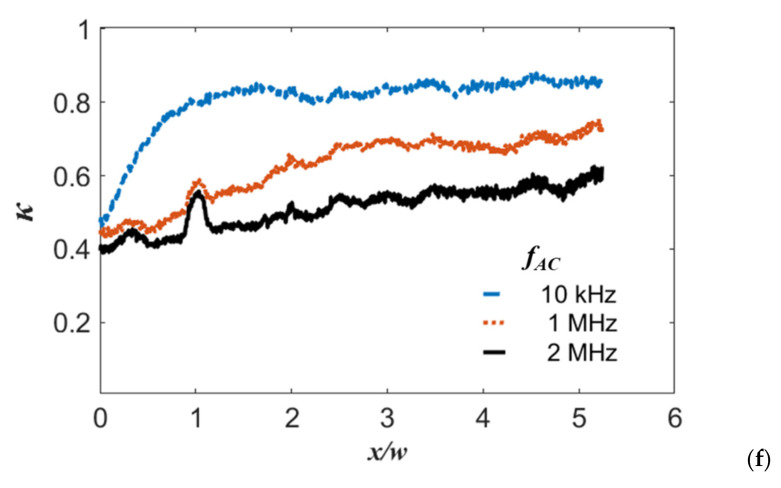
Visualization and comparison of mixing results in microchannels with different frequencies of AC and DC signals. Here, γ=10 and $E_{A}$ = 1000 V/cm. (**a**) DC, (**b**) $f_{AC}=$ 10 KHz, (**c**) $f_{AC}=$ 1 MHz, (**d**) $f_{AC}=$ 2 MHz, and (**e**) comparison of concentration profile in transverse direction at x=2.3w from the channel entrance with AC signal. (**f**) Mixing index varying along streamwise direction at different AC frequencies.

Figure 6 shows the results of mixing under DC voltage and different frequencies of AC voltage. When $f_{AC}=$ 10 KHz, the mixing performance is stronger than that when the frequencies are 1 MHz and 2 MHz. When DC voltage was applied on electrodes, strongest mixing was achieved in a very short time. However, bubbles were also generated within 1 s since voltage was applied. The channel was finally blocked by these bubbles. C distributions in the transverse direction are shown in Figure 6e for three different AC frequency mixing results. According to this quantitative C distribution, when $f_{AC}=$ 10 KHz, concentration distribution reaches relative uniformity at x=2.3w from the entrance. At the same streamwise position, however, when $f_{AC}=$ 1 MHz and 2 MHz, the profiles of the concentration distribution are still far from uniform. From Figure 6f, it can be seen that κ is always the largest at $f_{AC}=$ 10 KHz, compared with that under other AC frequencies. 

**Figure 7:**
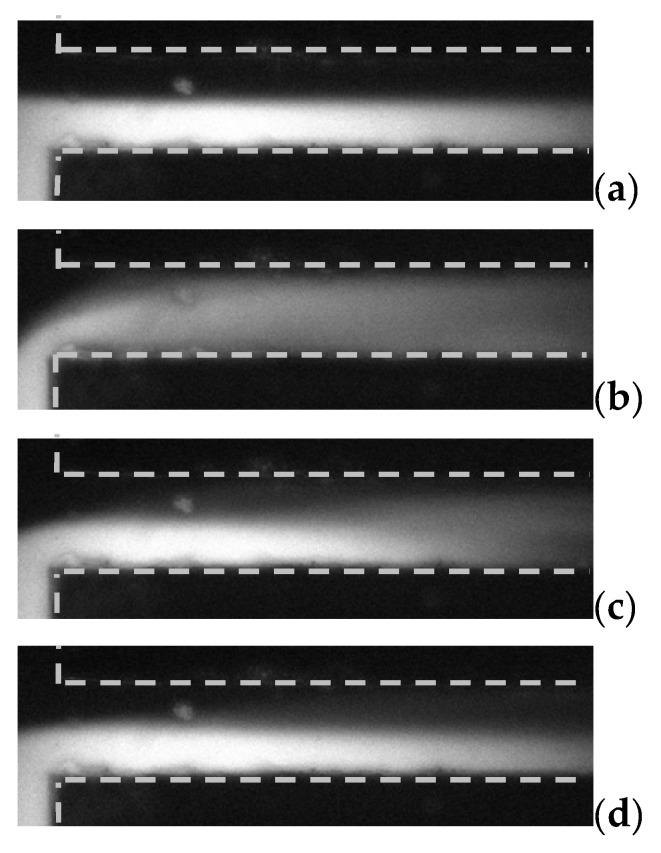
Visualization and comparison of mixing result in microchannels with different $f_{AC}$. Here, γ=10 and $E_{A}$ = 1667 V/cm. (**a**) No voltage, (**b**) 30 MHz, (**c**) 35 MHz, and (**d**) 40 MHz.

Moreover, mixing results under high frequency were also investigated. It was found that, rapid mixing result can be also achieved at high frequency besides low frequency as long as the $\vec{E}$ is sufficiently strong. Results are shown in Figure 7. Applied frequencies vary from 30 MHz to 40 MHz, and the $E_{A}$ was increased to 1667 V/cm. 

Figure 7 shows mixing results under each $f_{AC}$. It obviously shows that mixing is stronger when $f_{AC}=$ 30 MHz, than that when $f_{AC}=$ 35 MHz and 40 MHz. However, at $E_{A}$ = 1667 V/cm (limitation of the function generator), 40 MHz was the highest frequency at which we can achieve mixing augmentation in this mixer. It is also an important advantage that mixing can be acquired under high AC frequency electric field, since in many cases, low frequency AC signal could generate bubbles due to electrolysis in microchannels, especially when highly conductive buffer is used. The present new design of the micromixer could significantly reduce the risk of generation of bubbles in the microfluidic device, when the operation AC frequency is increased to higher than 10 kHz. Especially, even in fluids with relatively high conductivity (1000 µS/cm), no bubble was generated.

**Figure 8:**
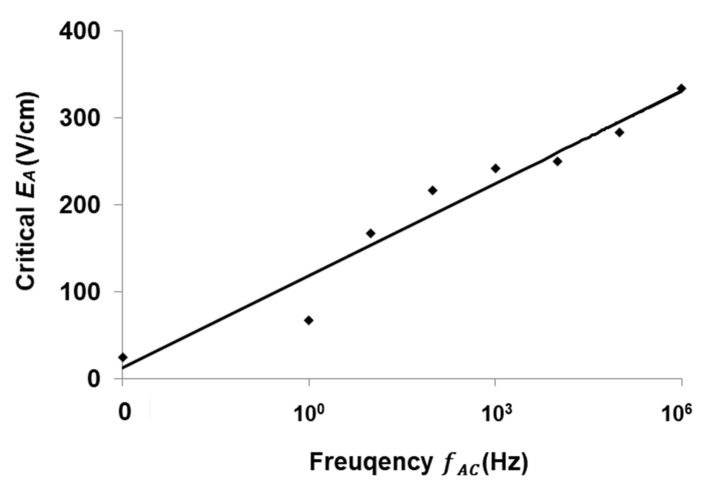
Critical electric field vs. the $f_{AC}$ for mixing enhancement. Flow rate was kept at 5 µL/min with γ=10.

It is known that the EK flow can become unstable or perturbated when applied $E_{A}$ exceeds a threshold value under a certain frequency. This particular $E_{A}$ value is called critical $E_{A}$, beyond which the interface becomes fluctuating. The relation between critical $E_{A}$ and frequency was investigated in the micromixer with conductive sidewalls, as plotted in Figure 8. 

Figure 8 suggests that, along with the increasing of frequency, the critical voltage required for the mixing enhancement is also increased. For $f_{AC}=$ 1 Hz, $E_{A}$ = 67 V/cm is sufficiently large to result in mixing augmentation inside the microchannel. When the frequency is increased to 1 MHz, $E_{A}$ = 333 V/cm is required to enhance the mixing. Since the applied AC frequency covers 6 orders, several different EK mechanisms could exist in the mixing process. Although the general form of electric body force is known, a comprehensive theory for predicting the critical values of local $E_{A}$ has not been established for the broad frequency range. Nevertheless, in the high frequency regime, i.e., $f_{vc}\ll f_{AC}\ll \langle \sigma \rangle /2\pi \varepsilon$ (where $f_{vc}$ is the cut-off frequency of velocity fluctuation in frequency domain), according to the theory of Zhao and Wang [13], we have approximately:

(5) $$\left|{\vec{f}}_{e}\right|\sim -\frac{\varepsilon E_{A}^{2}}{w}\left(\frac{\gamma -1}{\gamma +1}\right)\left(1-\beta^{2}\right)$$

where $\beta =2\pi f_{AC}\varepsilon /\langle \sigma \rangle$ is a dimensionless AC frequency. As $f_{AC}$ is increased, β increases accordingly, and thus, ${\vec{f}}_{e}$ is decreased. To generate sufficiently large ${\vec{f}}_{e}$ to disturb the flow, $E_{A}$ must accordingly increase simultaneously.

According to the theoretical research of Zhao and Wang [12,13], the electric volume force in DC electric field is larger than that in AC electric field, under equivalent electric field magnitudes. The present experimental investigation on frequency effect supports the theoretical conclusion, and the fastest mixing could be achieved under DC electric field in a very short time. However, for practical applications, to avoid bubbles generated by electrolysis, the AC electric field is applied.

### 3.4. Electric Field Effect

As the EBF plays a key role in the currently designed mixing process, mixing should be directly related to $\vec{E}$. Therefore, voltage effect on mixing result was investigated. In this experiment, frequencies of the applied signals are kept constant, i.e., $f_{AC}\;$= 10 kHz. $E_{A}$ was varied from 0 V_p-p_ to 1167 V/cm (14 V_p-p_).

**Figure 9:**
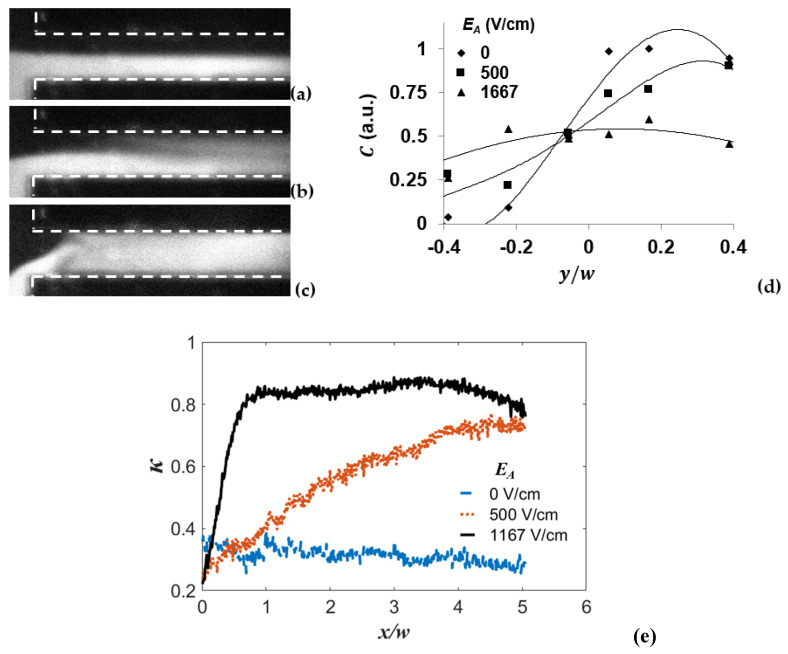
Visualization and comparison of mixing result in microchannels with various $E_{A}$. Here, $f_{AC}=$ 10 kHz and γ=10. (**a**) 0; (**b**) 500 V/cm; (**c**) 1167 V/cm; and (**d**) comparison of concentration profile in transverse direction at x/w=3 from the channel entrance with different AC voltages. (**e**) Mixing index varying along streamwise direction at different $E_{A}$.

Figure 9 shows mixing performance under different applied $E_{A}$. As visualized in Figure 9, despite the molecular diffusion, there is no obvious mixing on the interface of the two streams when no $E_{A}$ is supplied. However, the mixing can be significantly enhanced when the applied $E_{A}$ is increased to 500 V/cm. With further increasing $E_{A}$ to 1167 V/cm, the mixing becomes the strongest.

Figure 9d shows the quantitative concentration C distribution in the transverse direction, at streamwise position x/w=3 away from the entrance. It shows that, C distribution under 1167 V/cm reaches relative uniformity at x/w=3 from the entrance, while at the same streamwise position, the profile of the concentration distribution under $E_{A}$ of 0 and 500 V/cm are still far from flat. The same consequence can also be concluded from Figure 9e, where higher electric field results in higher mixing index. Especially at $E_{A}=$ 1167 V/cm, κ reaches 0.83 at x/w=0.71, which only costs time in the amount of 14 ms. All the results indicate that mixing is enhanced rapidly with increased electric field in the conductive sidewalls micromixer. 

It should be noted that, besides the EK flow generated directly on the interface of electric conductivity gradient, there are also two additional EK flows generated in the electrokinetic micromixer system. One is the induced charge electrokinetic flow adjacent to the electrodes [33], the other is electro-osmotic flow on the top and bottom walls. 

When the electric voltage applied is sufficiently large, nonlinear induced charge with vortical structures can be induced adjacent to the electrodes because of concentration polarization. The flow can be chaotic and apparently enhance fluid mixing in the diffusion layer, which is several hundred times of Debye length from electrodes [34]. Therefore, the mixing of fluids can be enhanced by nonlinear EK flow induced near electrodes. Besides, due to the unbalanced electric field on the low and high electric conductivity streams, a large scale vortical flow can be generated by the electro-osmotic flow (EOF) adjacent to the top and bottom walls, as have been investigated by Nan et al. [25]. The vortical flow could significantly enhance the 3D mixing of fluids on large scales. Thus, the fast mixing is achieved as a result of all these EK mechanisms.

### 3.5. Re Number Effect

**Figure 10:**
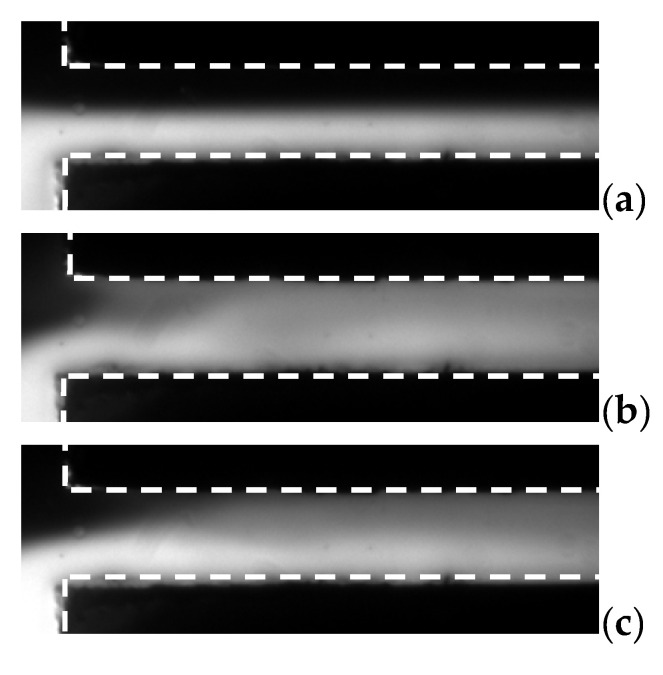

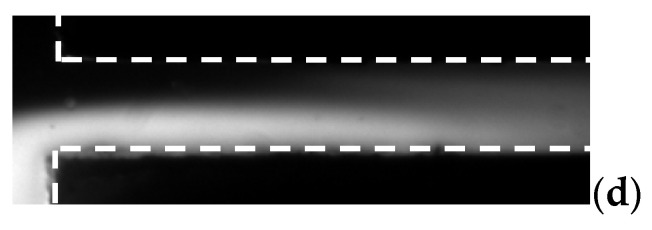
Visualization and comparison of mixing result in the micromixer with different Re numbers. (**a**) No voltage, (**b**) Re = 0.1, (**c**) Re = 0.3, and (**d**) Re = 0.5. Applied $E_{A}$ was kept at 500 V/cm with $f_{AC}=$ 10 kHz.

Different Re number effects on mixing results were investigated as well. In this experiment, frequency of the applied signal is kept constant, i.e., $f_{AC}=$ 10 kHz. Applied $E_{A}$ was kept at 500 V/cm. Flow rate was changed in the range of 1 µL/min to 5 µL/min to increase Re. Three different Re numbers, i.e., 0.1, 0.3, and 0.5 were compared. Results are shown in Figure 10.

As visualized in Figure 10, the mixing is strongest at a given downstream position when the Re number was 0.1, compared with situations where the Re numbers were 0.3 and 0.5. The mixing length (the downstream distance from the inlet of the channel required for the mixing to be achieved in the transverse direction, not the Prandtl mixing length in turbulent flows) is much shorter when low Re number was applied than when high Re number was applied. Note that although the mixing length is shorter at lower Re, the mixing time (required for the mixing to be achieved in transverse direction) is not necessarily shorter because the bulk flow velocity is larger in the higher Re. 

Here we use mass transport equation to explain the observed effect of Re on mixing. In EK micromixer, the mixing is dominated by the scalar transport due to velocity fluctuations. This can be explained by a convection–diffusion equation, as:

(6) $$\frac{\partial C}{\partial t}+\vec{u}\cdot \nabla C=D\nabla^{2}C$$

where D is the diffusion coefficient. Considering a quasi-steady process, i.e., $\vec{u}=\vec{u^{\prime }}+U$ (where $\vec{u\prime }$ is the velocity fluctuation primarily attributed to EBF), $C=\bar{C}+c^{\prime }$ and $\partial \bar{C}/\partial t=0$. $\bar{C}$ and $c^{\prime }$ are the mean value and fluctuations of concentration, respectively. Subsequently, we have:

(7) $$\frac{\partial c\prime }{\partial t}+\left(\vec{u^{\prime }}+U\right)\cdot \nabla \left(\bar{C}+c^{\prime }\right)=D\nabla^{2}\left(\bar{C}+c^{\prime }\right)$$

Taking temporal averaging on Equation (6), we have:

(8) $$\bar{\vec{u^{\prime }}\cdot \nabla c^{\prime }}+U\cdot \nabla \bar{C}=D\nabla^{2}\bar{C}$$

By combining Equations (6) and (7), and considering U is only in streamwise direction, we further have the transport equation of c′, which is:

(9) $$\frac{\partial c\prime }{\partial t}+\vec{u^{\prime }}\cdot \nabla \bar{C}+\vec{u^{\prime }}\cdot \nabla c^{\prime }+U\frac{\partial c^{\prime }}{\partial x}-\bar{\nabla \cdot \left(c^{\prime }\vec{u^{\prime }}\right)}=D\nabla^{2}c^{\prime }$$

Since EBF is perpendicular to the flow direction, $\vec{u^{\prime }}$ is in transverse direction initially at the interface between the two streams. Normally $c^{\prime }/\bar{C}\ll 1$, we dimensionally have $\vec{u^{\prime }}\cdot \nabla \bar{C}\gg \vec{u^{\prime }}\cdot \nabla c^{\prime }$. If we only focus on large-scale concentration fluctuations, the influence of the diffusion term can also be ignored. Thus, the initial spreading of the mixing can be approximately determined by:

(10) $$\frac{\partial c\prime }{\partial t}+v^{\prime }\frac{\partial \bar{C}}{\partial y}+U\frac{\partial c^{\prime }}{\partial x}-\bar{\nabla \cdot \left(c^{\prime }u^{\prime }\right)}=0$$

where $v^{\prime }$ is the velocity fluctuation component in y direction. The mixing of fluids is primarily determined by two convection terms, which are $v^{\prime }\partial \bar{C}/\partial y$ and $U\partial c^{\prime }/\partial x$. Dimensionally, in the EK flow, ${v^{\prime }}^{2}\sim \left|{\vec{f}}_{e}\right|$, and U~Re. When the electric field intensity and solutions are given, $\left|{\vec{f}}_{e}\right|$ could be approximately fixed in the initial stage in this investigation, and thus ${v^{\prime }}^{2}$ remains approximately unchanged. In addition, in turbulent flows, commonly,$\;\frac{v^{\prime }}{U}<1$. Consequently, as Re is increased, $U\partial c^{\prime }/\partial x$ convects and transports more mass downstream before they are spreading along transverse direction by the relatively smaller $v^{\prime }\partial \bar{C}/\partial y$. Hence, mixing in our mixer could have a much shorter mixing length under lower Re number than that under high Re number.

## 4. Conclusions

In this paper, a novel quasi T-channel micromixer with conductive sidewalls is introduced. Compared with the conventional micromixers, where electrodes are located at the ends of the channel and the electric field and conductivity gradient are orthogonal, the micromixer with conductive sidewalls, where the electric field and conductivity gradient are parallel, can generate faster mixing under the same electric field. In the present device, no amplifier or high voltage supply is required, and a function generator is sufficient to create fast mixing. Furthermore, effects of Re numbers, electric field strength, AC frequency, and conductivity ratio on mixing results have been studied in the conductive sidewall micromixer. The results reveal that the mixing length is shorter with lower Re number and AC frequency, and stronger electric field and higher conductivity ratio. This mixing strategy provides a new and convenient method for enhancing the mixing of two fluids at low Re in microchannels, which is a common key step in sample pretreatment in biomedical and biochemical analysis applications.
